# The effect of movement system impairment-based classification treatment compared to routine physiotherapy on pain, disability, alignment, and movement impairments in individuals with tibiofemoral rotation syndrome: a randomized controlled trial

**DOI:** 10.1186/s13102-024-00883-9

**Published:** 2024-04-25

**Authors:** Forouzan Mousavi, Mehrnaz Kajbafvala, Holakoo Mohsenifar, Reza Salehi, Anahita Hejazi

**Affiliations:** 1 Iranian Center of Excellence in Physiotherapy, Rehabilitation Research Center, Department of Physiotherapy, School of Rehabilitation Sciences, University of Medical Sciences, Tehran, Iran; 2 Department of Physiology, School of Medicine, Iran University of Medical Sciences, Tehran, Iran

**Keywords:** Classification, Knee, Physical therapy, Rehabilitation

## Abstract

**Background:**

Knee pain is a common musculoskeletal problem. Lower extremity movement impairments could alter stresses in different planes and contribute to knee pain. Classifying these impairments may be helpful in the diagnosis and treatment of knee problems. Movement system impairment (MSI)-based classification is a system to evaluate movement impairments. Trials that involve this classification are limited. Therefore, it will be of interest to examine the effect of movement system impairment-based classification treatment compared to routine physiotherapy in individuals with tibiofemoral rotation syndrome.

**Methods:**

Twenty-two individuals with knee pain aged 18–40 years (2 males, 20 females) diagnosed with tibiofemoral rotation (TFR) syndrome were included. After initial evaluation, individuals were randomly assigned into two treatment groups (MSI-based treatment and routine physiotherapy). Both treatment groups contained 8 treatment sessions over 4 weeks. Alignment and movement impairments data form, a numeric rating scale (NRS), and the Kujala Disability Questionnaire were assessed at baseline and after a four-week intervention. Independent samples t-test and Mann-Whitney U test were used for quantitative variables, and Fisher’s exact test was employed for qualitative variables to compare the groups. One-way Analysis of variance (ANOVA) and paired samples t-test were utilized to within-group changes of quantitative variables, and qualitative variables were analyzed with the McNemar test.

**Results:**

The results showed that pain intensity and disability significantly decreased within and between groups after intervention (*P* > 0.05). There were also statistically significant differences between treatment groups for 3 out of 6 alignment and movement impairments (PS-FAdd/IR, Step down-Add/Valgus, and STS-Add/Valgus) (*P* > 0.05). Within-group differences for alignment and movement impairments were significant only for the MSI-based treatment group (*P* > 0.05).

**Conclusions:**

The findings suggest that a specific MSI-based treatment, considering a homogenous group of individuals with knee pain, may contribute to pain, disability, and alignment and movement impairments improvement. Therefore, it is important to notice the classification-based treatment for individuals with knee pain.

**Trial Registration Number (TRN) and date of registration:**

The trial was registered at the (https://www.irct.ir), (IRCT20210505051181N3) on 17/7/2021.

**Supplementary Information:**

The online version contains supplementary material available at 10.1186/s13102-024-00883-9.

## Introduction

Lower extremity movement impairments have been proposed to alter stresses in the frontal, sagittal, and transverse planes [1–3] and contribute to knee pain and other common musculoskeletal problems such as patellofemoral pain (PFP) [4–6], anterior cruciate ligament (ACL) tear [7], and knee osteoarthritis (OA) [8, 9]. Movement impairments may exist as an abnormal alignment or altered movement pattern during activities of daily living [2, 10]. Identifying and categorizing these impairments may be essential and helpful in directing the appropriate treatment protocols [2, 10, 11].

Different conservative treatments have been mostly recommended for people with knee problems [12–16]. However, the literature represents conflicting results regarding the effectiveness of various conservative care [10, 17]. A possible explanation for the lack of consistent evidence could be sample heterogeneity. Therefore, to enhance the treatment effect, assigning to more homogenous subgroups may lead to specific diagnoses and treatment for each subgroup [18–20].

Movement system impairment (MSI)-based classification is one of the classification systems available to assess human movement system impairments [2, 21]. The MSI classification is based on clusters of signs and symptoms developed to categorize patients into homogenous subgroups through a standardized clinical examination to guide physical therapy interventions and to inform prognosis [19, 20, 22, 23]. The physical examination leads to classifying people with knee pain into one of seven possible knee MSI subgroups that are named regarding the alignments or movement patterns that reproduce the patient’s symptoms [21, 24]. Tibiofemoral rotation (TFR) syndrome, as one of the knee MSI categories, is distinguished by knee pain associated with impaired tibiofemoral rotation. It could be characterized by varus (TFRVar) or valgus (TFRVal). TFRVal is the most common TFR syndrome, and women are more likely to show TFRVal than men [2]. TFR syndrome is diagnosed with pain along the tibiofemoral joint line, around the patella, or at the junction of the iliotibial band (ITB). Activities involving rotation between the tibia and femur, such as weight-bearing (running) or non-weight-bearing activities (sitting) could often be associated with the pain [2].

The treatment based on the MSI classification consisted of specific exercise prescriptions and patient education to correct impairments of alignment and movement associated with knee pain during functional activities [2]. The primary aim of treatment is to instruct the patients in correcting the performance of functional activities and doing home exercises. Emphasizing the training of patients to restrict tibiofemoral rotation during functional activities is crucial. This approach helps prevent the recurrence of preferred movement and alignment strategies that may cause injury and pain, and it promotes adherence to the treatment program [2, 10].

Several studies are reporting the reliability of the MSI classification [11, 19, 20, 22]. The reliability and validity of the knee MSI classification system have been reported in our previous studies. However, to the best of the authors’ knowledge, a clinical trial comparing treatment regarding the MSI classification with routine physiotherapy has not yet been investigated. There is only one case report that is devoted to examining a patient with knee pain based on the MSI classification system [10]. Therefore, this study aimed to compare the effect of movement system impairment-based classification treatment to routine physiotherapy in individuals with tibiofemoral rotation syndrome. This is so that if significant changes are observed due to the treatment based on the MSI classification compared to routine physiotherapy, treatment based on the MSI classification could be used in the clinical setting.

## Methods

### Trial design

**Fig. 1:**
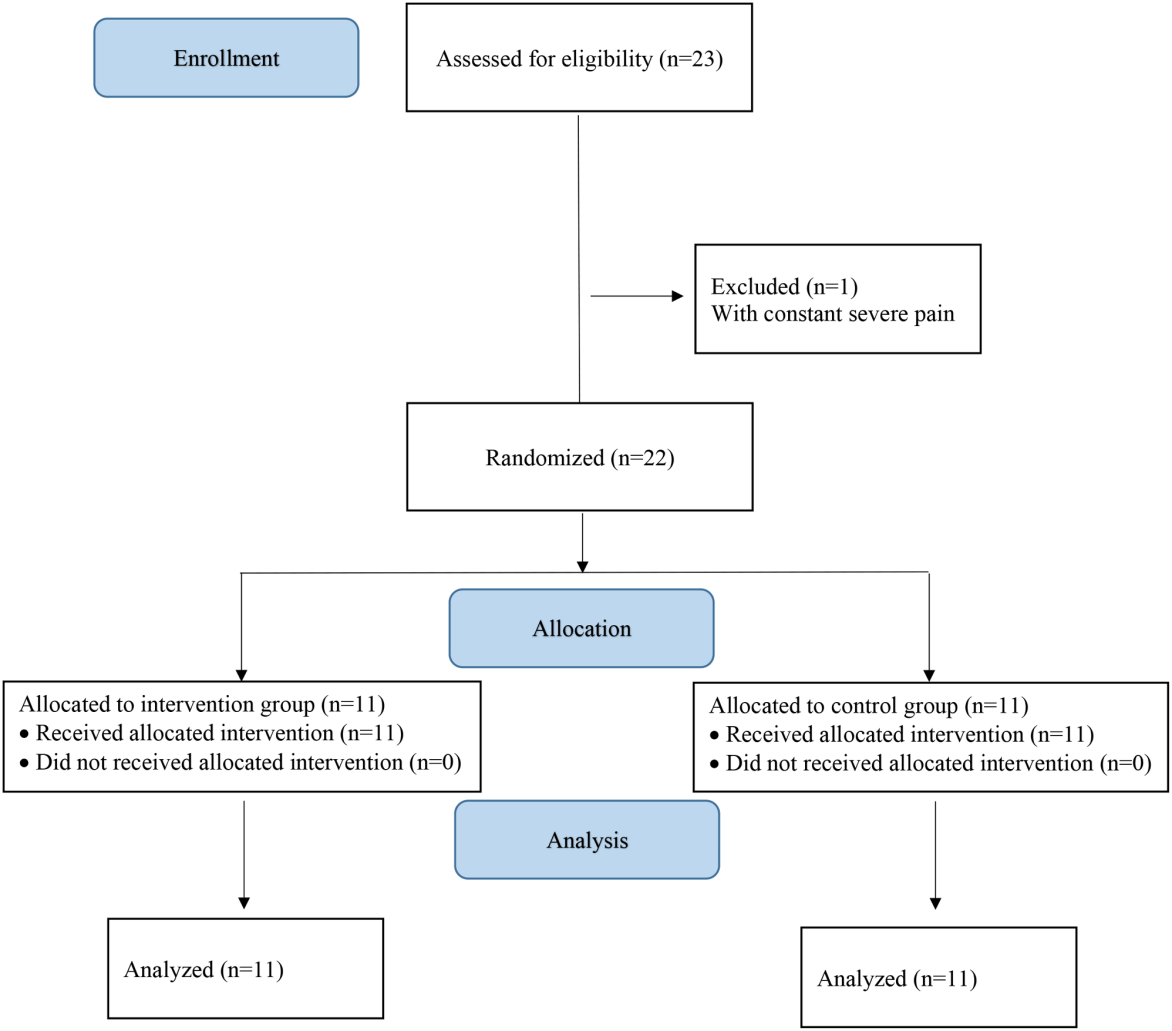
The CONSORT flow chart of participants

This study was designed as a single-center, randomized, single-blinded, controlled trial with a parallel group of 22 patients. The allocation ratio was 1:1. This study followed the CONSORT guidelines, checklist (supplementaryS1 1), and flow chart (Fig. 1). You can find more details regarding the protocol of this trial at https://www.irct.ir with the IRCT20210505051181N3 reference number.

### Participants

Twenty-two adults with knee pain diagnosed with TFR syndrome were recruited through advertisements as a sample of convenience. Demographic variables, including age, gender, weight, and height were recorded. BMI was obtained from height and weight measurements (weight divided by height squared). This study was conducted in the physiotherapy clinic of the School of Rehabilitation Sciences, Iran University of Medical Sciences, Tehran, Iran from March 2022 to October 2022. Subjects were included if they had the following inclusion criteria: (1) age between 18 and 40 years [25–27],2) history of sudden or gradual pain at the knee complex or surrounding tissues [28, 29],3) a pain intensity of at least 3 points on a numerical pain rating scale (NPRS) [29, 30].4) classified as TFR syndrome on initial assessment by using the physical examination form [2, 10]. Subjects were excluded if they had: 1) any structural deformities of the spine and lower extremities, 2) pregnancy, 3) diabetes, 4) use of assistive device, 5) a history of knee surgery in the last three months, 6) a history of more than one surgical operation on the knee, 7) constant severe pain, 8) used analgesic and anti-inflammatory drugs at the time of study. In addition, known cases of cancer, lumbosacral radiculopathy, neuromuscular disorders, rheumatoid arthritis, and cardiopulmonary disease. All subjects signed an informed consent regarding the process of examination. The study was approved by the Ethical Committee of the Iran University of Medical Sciences.

#### Initial assessment and TFR syndrome diagnosis

The initial assessment consisted of evaluating the symptom and sign items in the physical examination form (Supplementary S2) and recording findings. In the symptom items, the patient’s responses to different test positions or movements were specified. If the given test aggravates the patient’s symptoms, the patient is asked to perform a modified test, involving the same test with correction of the observed impairments. For the sign items, the patient’s alignments and movement patterns were observed by the examiner. Finally, the examiner made a judgment based on the MSI classification [2]. Patients who were classified as TFR syndrome on initial assessment were considered eligible for the study. Excellent inter-rater reliability for the symptom items (kappa values ranged from 0.83 to 1.00), poor to excellent inter-rater reliability for the sign items (kappa values ranged from 0.18 to 1), and poor to excellent intra-rater reliability for the symptom and sign items (kappa values ranged from 0.00 to 0.83, and 0.00 to 0.82, respectively [31, 32]) of the knee MSI classification were reported previously [32].

#### Interventions

The participants were randomly assigned to experimental or control groups, which received either MSI-based treatment plus basic electrotherapy (*n* = 11) or routine physiotherapy plus basic electrotherapy (*n* = 11). Both groups were treated by a trained and experienced physiotherapist (FM) with more than 3 years of clinical experience in managing patients with musculoskeletal problems. She passed a 10-hour practical course to master the details of the physical examination process based on the MSI classification system. The treatment session commenced 30 min after the first examination. Both groups (MSI-based treatment and routine physiotherapy) consisted of 8 treatment sessions over 4 weeks (2 sessions per week). The duration of entire treatment session lasted 60 min.

### Basic electrotherapy treatment

Participants in both groups received conventional Transcutaneous Electrical Nerve Stimulation (TENS) at the beginning of each session on the knee for 20 min at a frequency of 120 HZ and a duration of 50 µs [33, 34], and pulse ultrasound with an intensity of 0.1 W/cm2 and a duty cycle of 20% [33–38].

### Routine physiotherapy (control group)

**Fig. 2:**
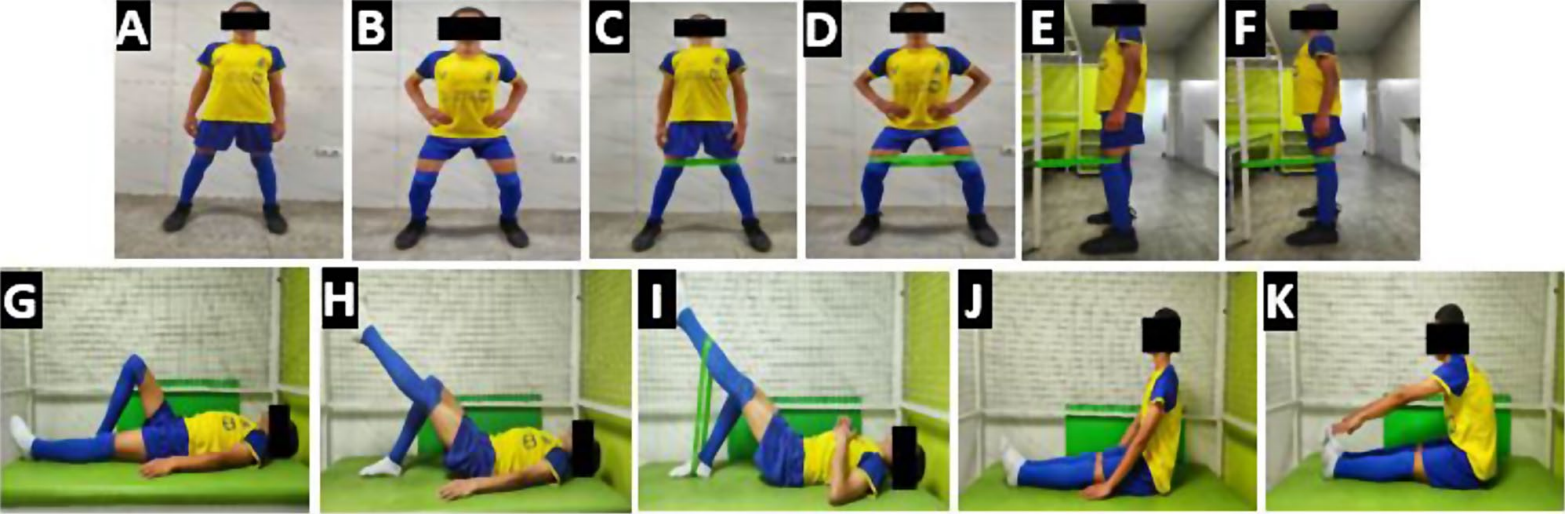
Routine physiotherapy exercises (**A**), (**B**), (**C**), (**D**) partial squatting, (**E**), (**F**) Terminal knee extension, (**G**), (**H**), (**I**) SLR, (**J**), (**K**) Hamstring & gastrocnemius stretching

Participants in the control group received routine physiotherapy based on the guideline to improve the function of the quadriceps muscle and to stretch the muscles adjacent to the knee joint [39]. The stretching exercises addressed the lower extremity muscles [14, 40, 41]. In addition, each patient performed strengthening exercises for lower extremity muscles, especially the quadriceps [29, 42, 43] (Fig. 2). A daily home exercise program with a photograph, written description, and instructions was provided to each patient. Supplementary 3 lists the specific details of the treatment protocol and progression of exercises for the routine physiotherapy group.

#### MSI-based treatment (experimental group)

**Fig. 3:**
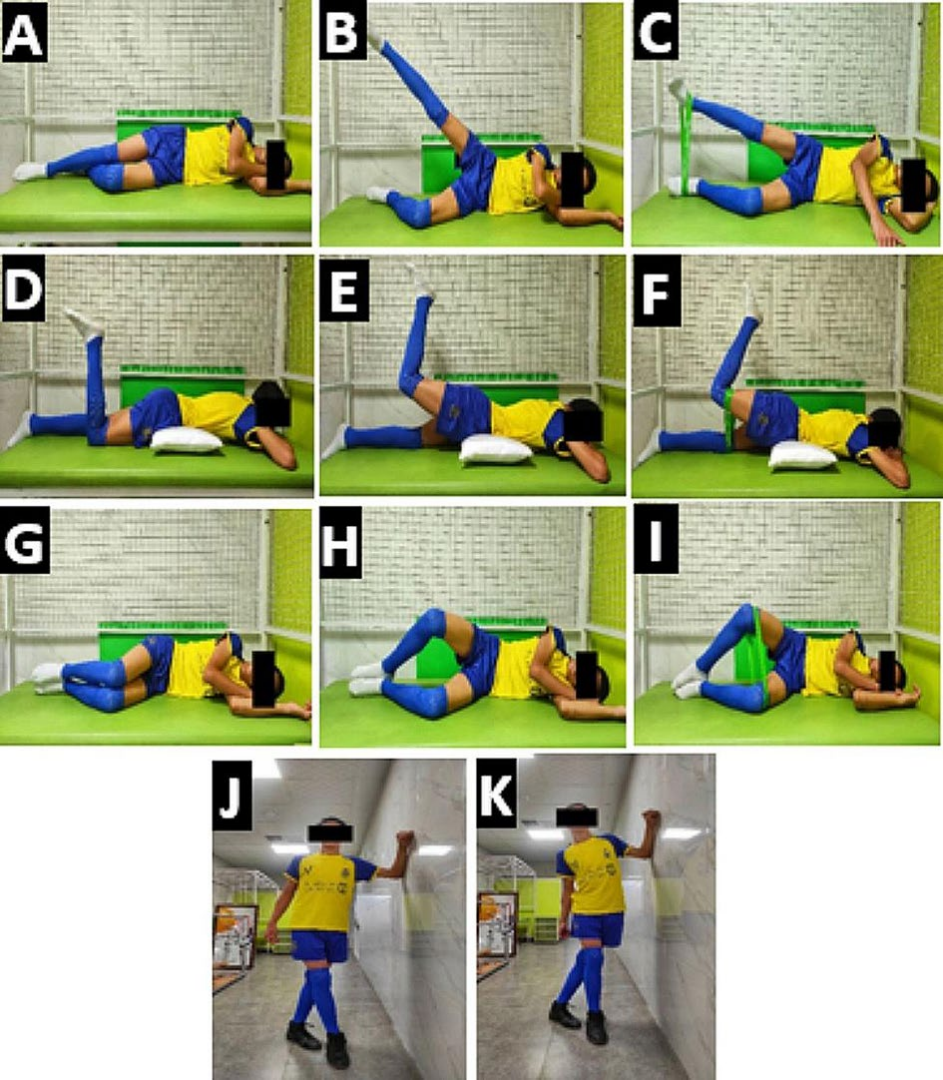
MSI-based exercises (**A**), (**B**), (**C**) Side SLR, (**D**), (**E**), (**F**) Prone hip extension, (**G**), (**H**), (**I**) Clamshell exercise, (**J**), (**K**) ITB stretching.

Treatment based on the MSI classification system included: 1) patient education, 2) modification and correction of performance of daily activities, and 3) prescription of specific flexibility and strengthening exercises. Patients were educated regarding the control and correction of abnormal alignments and altered movement patterns related to the TFR syndrome. In addition, patients were warned about the repetition of sustained postures and repeated movements that were associated with a specific direction. Patient education was continued during all treatment sessions. The performance of daily activities was assessed and the activities that provoked the patient’s symptoms were identified. In the following, any corrections and modifications were guided by the physiotherapist during all treatment sessions. The specific exercises were prescribed to correct the identified alignment and movement impairments. Also, lower extremity flexibility and strengthening exercises related to the TFR syndrome were addressed (Fig. 3). Patients were also advised to do home exercise [2, 10]. Specific details of the treatment protocol and progression of exercises for the MSI-based treatment group are provided in Supplementary S4.

### Outcomes

The outcome measurements were performed by an assessor (FM) before the commencement of the first treatment session and after the end of the eighth session (2 times of evaluation in total).

### Primary outcome measures

#### Pain intensity and disability

In the current study, pain and disability were the primary outcomes. NPRS, and the Kujala Disability Questionnaire, were used to assess pain intensity and disability level. The NPRS measures pain intensity, in which patients rate pain intensity from 0 (no pain) to 10 points (worst imaginable pain) [44]. The participants were asked to rate their pain intensity during the past 24 h plus the previous week [44]. The Kujala Disability Questionnaire was utilized to assess disability level associated with knee pain [45–48]. It has 13 self-report items describing functional activities such as walking, running, jumping, squatting, stepping up/ down, and prolonged sitting with a bent knee. Higher scores indicated lower disability [49]. The Persian version of the Kujala Disability Questionnaire is a reliable and valid assessment tool [49].

### Secondary outcome measure

#### Alignment and movement impairments

The alignment and movement impairments were considered as the secondary outcome measure in this trial that were evaluated by the valid data form used for the visual assessment of the patient’s following signs: 1) Femoral adduction/ internal rotation during partial squat (PS-FAdd/IR), 2) Knee valgus during partial squat (PS-Valgus), 3) Tibia external rotation/ abduction during two joint hip flexor length test (TJHFLT-TibiaER/Abd), 4) Tibia external rotation during step-up (Step up-TibiaER), 5) Femoral adduction/ knee valgus during step-down (Step down-Add/Valgus), 6) Femoral adduction/ knee valgus during sit to stand (STS-Add/Valgus) (Supplementary S5) [31].

### Sample size

The required sample size was based on the information obtained from the preliminary study (on 10 patients) with a statistical power of 0.8 and α of 0.05, by adding a 10% drop-out rate, 22 people were calculated.

### Randomization

The randomization process was done by a clinic secretory, outside the research team, before the start of the study. Following the initial assessment, the eligible patients were assigned to either the MSI-based treatment or routine physiotherapy with an allocation ratio of 1;1, using block balanced randomization method. Random allocation was done by the method of variable blocks, which includes 1-letter blocks, made of letters A and B. The obtained treatment assignment was placed in numbered envelopes in the form of letters A and B. The sealed numbered envelopes, related to the sequential number of each person, were presented and the therapeutic interventions were adjusted regarding the letters inside the envelopes. The examiner and participants were unaware of the letters until the end of the study.

### Blinding

In this single-blind study, the patients were unaware of the specific allocation and treatment group assignment.

### Statistical analysis

Statistical analyses were performed by using SPSS, version 27.0 (SPSS Inc., Chicago, IL). The Shapiro-Wilk test was used to check the distribution of quantitative variables. Independent sample t-test and Mann-Whitney U test were used for quantitative variables, and Fisher’s exact test was used for qualitative variables to compare groups at baseline and after interventions. Within-group changes in quantitative variables were analyzed with paired samples t-test and One-way ANOVA, and qualitative variables were analyzed with the McNemar test. It should be noted that the data are expressed as mean ± SD and P value < 0.05 was considered significant statistically.

## Results

The baseline demographic characteristics of the participants are shown in Table 1. There were no significant differences between the two treatment groups regarding the baseline demographic characteristics and outcome measures (*P* > 0.05). The results demonstrated significant differences between treatment groups for the primary outcome measures of pain (mean difference = -1.27, 95% CI = (0.14–2.40), and disability (mean difference = 7.46, 95% CI = (-13.37 - -1.54) (Table 2). In addition, pain and disability significantly decreased post-intervention versus pre-intervention in both groups (Table 3). There were also statistically significant differences between treatment groups for three out of six alignment and movement impairments (PS-FAdd/IR, Step down-Add/Valgus, and STS-Add/Valgus), as the secondary outcome (*P* < 0.05) (Table 4). There were no significant differences for alignment and movement impairments pre- and post-intervention in the routine physiotherapy group (*P* > 0.05) (Table 5). Four out of six alignment and movement impairments had significant differences pre- and post-intervention in the MSI group (*P* < 0.05) (Table 5).

**Table 1:** Demographic characteristics of the subjects

Variable (Unit)	Routine physiotherapy (*n* = 11)	MSI-based treatment (*n* = 11)
Age (y)	31.47 (3.65)	32.30 (4.90)
Sex Male Female	1 (9.09%) 10 (90.90%)	1 (9.09%) 10 (90.90%)
Weight (kg)	64.82 (13.82)	69.82 (9.13)
Height (cm)	165.40 (6.08)	163.70 (4.31)
Body Mass Index (kg/m2)	23.87 (5.95)	27.11 (4.11)

**Table 2:** Mean (SD) of pain intensity, and disability and comparison between group

Outcomes	Measurement time	Routine physiotherapy	MSI-based treatment	Between-group differences	
		*Mean (SD)*	*Mean (SD)*	***P-value***	*Effect Size* (95% CI)
Pain intensity (0–10)	1 Week before Intervention	5.81 (1.53)	6.18 (1.32)	0.758	0.689 (-1.64-0.91)
	Baseline	4.90 (1.04)	5.00 (1.09)	0.877	0/094 (-0.09-0.45)
	After intervention	2.27 (1.34)	1.00 (1.18)	0.022	1.006 (0.14–2.40)
Disability (0-100)	Baseline	74.73 (6.97)	71.18 (8.97)	0.313	0.442 (-3.60-10.69)
	After intervention	82.36 (5.46)	89.82 (7.65)	0.016	1.123 (-13.37- -1.54)

**Table 3:** Within-group differences for the outcomes of pain intensity, and disability

Outcomes	Measurement time	Routine physiotherapy			MSI-based treatment		
		Mean difference	P-value	Effect Size (95% CI)	Mean difference	P-value	Effect size (95% CI)
Pain intensity	Baseline vs. 1 Week before Intervention	-0.90	0.497	0.696 (-0.50-2.32)	-1.18	0.602	0.975 (0.03–2.32)
	After intervention vs. 1 Week before Intervention	-3.54	0.002	2.462 (2.08-5.00)	-5.18	< 0.0001	4.138 (3.97–6.39)
	After intervention vs. Baseline	-2.63	0.023	2.193 (2.05–3.21)	-4.00	0.012	3.521 (2.98–5.02)
Disability	After intervention vs. Baseline	9.81	< 0.0001	1.393 (7.92–11.71)	18.64	< 0.0001	2.236 (14.82–22.45)

**Table 4:** Fisher’s exact test for the outcome of alignment and movement impairment

**Outcome**	**Group**	**Subject without impairment**	**Subject with impairment**	***P-value***
PS-FAdd/IR	Routine physiotherapy MSI-based treatment	2 8	9 3	*P* = 0.030
PS-Valgus	Routine physiotherapy MSI-based treatment	9 10	2 1	*P* > 0.999
TJHFLT-tibiaER/Abd	Routine physiotherapy MSI-based treatment	1 1	10 10	*P* > 0.999
Step up-tibiaER	Routine physiotherapy MSI-based treatment	2 4	9 7	*P* = 0.635
Step down-add/valgus	Routine physiotherapy MSI-based treatment	2 10	9 1	*P* = 0.001
STS-add/valgus	Routine physiotherapy MSI-based treatment	5 11	6 0	*P* = 0.012

**Table 5:** McNemar’s test for the outcome of alignment and movement impairments in each group

**Outcome**	**Group**	**Subject without Impairment**		**Subject with Impairment**		***P-value***
		**Pre-intervention**	**Post-intervention**	**Pre-intervention**	**Post-intervention**	
PS-FAdd/IR	Routine Physiotherapy	1	2	10	9	> 0.999
	MSI-based Treatment	0	8	11	3	0.008
PS-Valgus	Routine Physiotherapy	6	9	5	3	0.250
	MSI-based Treatment	0	10	11	1	0.002
TJHFLT-TibiaER/Abd	Routine Physiotherapy	0	1	11	10	> 0.999
	MSI-based Treatment	0	1	11	10	0.250
Step up-TibiaER	Routine Physiotherapy	1	2	10	9	> 0.999
	MSI-based Treatment	1	4	10	7	> 0.999
Step down-Add/Valgus	Routine Physiotherapy	1	2	10	9	> 0.999
	MSI-based Treatment	0	10	11	1	0.002
STS-Add/Valgus	Routine Physiotherapy	1	5	10	6	0.125
	MSI-based Treatment	2	11	9	0	0.004

## Discussion

This study was a randomized controlled trial assessing the effect of movement system impairment-based classification treatment compared to routine physiotherapy on pain, disability, alignment, and movement impairments in individuals with tibiofemoral rotation syndrome. The results showed significant differences in pain and disability between groups. In addition, significant differences in three out of six alignment and movement impairments were observed between groups after intervention.

In this study, pain and disability significantly decreased between and within groups. The MSI-based treatment group showed larger mean differences and effect sizes compared to the routine physiotherapy group. Considering that both groups had received exercise training, decrement in pain and disability was expected. According to the results of Clark et al., exercise training and hamstring stretching could reduce pain in people with knee pain [14]. Furthermore, the effect of the time on symptom improvement should be considered. Based on the time needed to achieve tissue healing, it is expected that pain will be resolved within 4 to 6 weeks according to Hayes et al. [10]. The authors hypothesized that the significant differences in pain and disability between groups could be attributed to patient education. This education focused on correcting abnormal alignments and altered movement patterns during functional activities, as well as strengthening and stretching exercises aimed at these alterations in the intervention group. Hayes et al. reported that the MSI-based treatment improved the patient’s pain and ability to perform functional activities [10]. The present study found different results for the alignment and movement impairments between and within groups. The MSI-based treatment group significantly improved PS-FAdd/IR, Step down-Add/Valgus, and STS-Add/Valgus compared to the routine physiotherapy group and pre- and post-intervention. These results would be due to the special focus of this approach on correcting femoral adduction/ internal rotation, and knee valgus by educating the patient on how to modify and control these impairments. The patient was advised to avoid bringing the knee to the midline during step down while contracting gluteal muscles during weight bearing phase to control excessive femoral internal rotation. Additionally, the patient was educated to prevent excessive femoral adduction and knee valgus during activity daily living. This included correcting the sit-to-stand movement pattern by maintaining the knee alignment with the second toe to avoid knee valgus when standing up. Some specific exercises including side SLR, clamshell, and TFL-ITB stretching to improve muscle function and flexibility were prescribed.

The authors hypothesized that the lack of difference in PS-Valgus, TJHFLT-TibiaER/Abd, and Step up-TibiaER between groups could be related to the similarities found in both treatment protocols and the fact that individuals in both groups adhered more to that were similar in both treatment programs. Both treatment protocols had a partial squatting exercise in which the patient maintained the alignment of the knee with the second toe and avoided bringing the knees together during exercise. Based on the evidence, additional therapeutic interventions could be used to correct the lower extremity rotational impairments such as a taping technique in which tibia external rotation could be sorely controlled and decrease patients’ symptoms [10]. Moreover, any alterations in alignments and movements of the ankle and foot structure could affect the tibiofemoral joint [50–53]. Therefore, addressing these impairments could alleviate symptoms and signs in individuals with knee pain.

The present study can be criticized for some limitations. First, individuals included in the study were recruited from the physical therapy clinic at the Rehabilitation School of Iran University of Medical Sciences. Therefore, they did not represent all the patients with knee problems. Furthermore, most individuals had mild to moderate knee pain. The results might be different if subjects with severe pain were included. Second, because of possible severe degenerative changes or some age-related motor control disorders patients older than 40 years were excluded from the study. Therefore, the results cannot be generalized to older adults. Third, our study had a 4-week exercise intervention, and outcomes were immediately measured after the intervention. Future studies should investigate a longer intervention period and a long-term follow-up. Fourth, due to the condition of the study, it was not possible to blind the treating physiotherapist to the assessment process.

## Conclusion

The results of this study indicated that individuals with tibiofemoral rotation syndrome had greater improvement in pain, and disability by receiving the MSI-based treatment compared to the routine physiotherapy. The results of the alignment and movement impairments showed that the MSI-based treatment improved PS-FAdd/IR, Step down-Add/Valgus, and STS-Add/Valgus. Also, none of the two treatment groups could be effective in correcting PS-Valgus, TJHFLT-TibiaER/Abd, and Step up-TibiaER. According to the study findings, treatment based on the MSI classification can be recommended among treatment priorities. The novelty of the study was using the classification-based treatment and comparing it with routine physiotherapy in patients with knee pain.

### Electronic supplementary material

Below is the link to the electronic supplementary material.

Supplementary Material 1

Supplementary Material 2

## Data Availability

No datasets were generated or analysed during the current study.

## Abbreviations

ACL: Anterior cruciate ligament
ANOVA: Analysis of variance
BMI: Body mass index
ITB: Iliotibial band
MSI: Movement system impairment
OA: Osteoarthritis
PFP: Patellofemoral pain
PS-FAdd/IR: Femoral adduction/ internal rotation during partial squat
PS-Valgus: Knee valgus during the partial squat
STS-Add/Valgus: Femoral adduction/ knee valgus during sit to stand
Step down-Add/Valgus: Femoral adduction/ knee valgus during step-down
Step up-TibiaER: Tibia external rotation during step-up
TFR: Tibiofemoral rotation
TJHFLT-TibiaER/Abd: Tibia external rotation/ abduction during two joint hip flexor length test
VAS: Visual analog scale
