# Supplementary material for: The effect of movement system impairment-based classification treatment compared to routine physiotherapy on pain, disability, alignment, and movement impairments in individuals with tibiofemoral rotation syndrome: a randomized controlled trial
Source: BMC Sports Sci Med Rehabil. 2024 Apr 25;16:94. doi: 10.1186/s13102-024-00883-9 (PMC11046785; doi:10.1186/s13102-024-00883-9)
Supplement: Supplementary file 2 — Supplementary Material 2 [file 13102_2024_883_MOESM2_ESM.docx]

**Supplementary 2: Physical Examination Form**

|  | | | |
| --- | --- | --- | --- |
| **Physical Examination Item (Signs)** | **Unit of Measurement** | | |
| ***Standing*** | |  |  |
| Alignment-anterior & posterior view | | Yes | No |
| Knee valgus | | Yes | No |
| Knee varus | | Yes | No |
| Femur internal rotation/adduction | | Yes | No |
| Tibia external rotation | | Yes | No |
| Foot supination | | Yes | No |
| Foot pronation | | Yes | No |
| Alignment-lateral view | | Yes | No |
| Knee flexion | | Yes | No |
| Knee hyperextension (extension > 5) | | Yes | No |
| Swayback with posterior pelvic tilt | | Yes | No |
| Increased lumbar curve | | Yes | No |
| Femoral internal rotation with single leg stance on involved limb | | Yes | No |
| Excessive ankle pronation with single leg stance on involved limb | | Yes | No |
| Tibia external rotation of involved leg with single leg stance on uninvolved limb | | Yes | No |
| Balance with single leg stance (Pelvic tilt/Trunk lateral bending with single leg stance) | | Yes | No |
| Femoral adduction/internal rotation with partial squat | | Yes | No |
| Knee valgus with partial squat | | Yes | No |
| Shift body weight posterior with partial squat | | Yes | No |
| Keeping tibia perpendicular to the floor with partial squat | | Yes | No |
| ***Sitting*** | | Yes | No |
| Lateral movement of patella with knee extension | | Yes | No |
| Relative flexibility with knee extension | | Yes | No |
| ***Supine*** | | Yes | No |
| Tibia external rotation/abduction with two joint hip flexor length test | | Yes | No |
| Relative flexibility with two joint hip flexor length test | | Yes | No |
| Relative flexibility with knee flexor length test | | Yes | No |
| ***Side*** | | Yes | No |
| Relative flexibility with Hip flexor length test | | Yes | No |
| ***Prone*** | | Yes | No |
| Tibia external rotation with knee flexion | | Yes | No |
| Relative flexibility with knee flexion | | Yes | No |
| Relative flexibility with hip rotation | | Yes | No |
| ***Functional activity*** | | Yes | No |
| Tibia external rotation of the swing leg with step up | | Yes | No |
| Pulling the knee back to the body with step up | | Yes | No |
| Femoral adduction and knee valgus with step down | | Yes | No |
| Excessive femoral internal rotation with walking | | Yes | No |
| Sitting with tibia in external rotation | | Yes | No |
| Femoral adduction and knee valgus when rising from sitting to standing | | Yes | No |
| Tibia external rotation when rising from sitting to standing | | Yes | No |

|  | | |
| --- | --- | --- |
| **Unit of Measurement** | **Physical Examination Item (Symptoms)** | |
|  | ***Standing*** | |
| Yes, no | Symptom in standing | |
| same, decreased, increased | Single leg stance on involved limb | |
| same, decreased, increased | Single leg stance on uninvolved limb | |
| same, decreased, increased | Partial squat | |
| same, decreased, increased | Corrected partial squat | |
|  | ***Sitting*** | |
| same, decreased, increased | Knee extension in sitting | |
| same, decreased, increased | McConnell test | |
| same, decreased, increased | Correction of McConnell test | |
|  | ***Supine*** | |
| same, decreased, increased | Two joint hip flexor length test | |
| same, decreased, increased | Corrected Two joint hip flexor length test | |
|  | ***Prone*** | |
| same, decreased, increased | Knee flexion in prone | |
| same, decreased, increased | Corrected Knee flexion in prone | |
|  | ***Functional activity*** | |
| same, decreased, increased | Sitting (specific to work) | |
| same, decreased, increased | Stairs | |
| same, decreased, increased | Gait | |
| same, decreased, increased | Rise from sitting to standing | |
|  | ***Muscle performance*** | |
| 0/5, 1/5, 2/5, 3/5, 4/5, 5/5 | Hip lateral rotator | |
| 0/5, 1/5, 2/5, 3/5, 4/5, 5/5 | Gluteus max | |
| 0/5, 1/5, 2/5, 3/5, 4/5, 5/5 | Posterior fiber of gluteus med | |
| 0/5, 1/5, 2/5, 3/5, 4/5, 5/5 | Iliopsoas | |
| 0/5, 1/5, 2/5, 3/5, 4/5, 5/5 | Hamstring | |
| 0/5, 1/5, 2/5, 3/5, 4/5, 5/5 | Quadriceps | |

**Supplementary 3: Treatment protocol and progression of exercises for the routine physiotherapy group**

| **Repetition/second** | **sets** | **Routine physiotherapy exercises** | **Week** |
| --- | --- | --- | --- |
| 10 Rep.  10 Rep.  10 Rep.  30 second | 3  3  3  3 | SLR  Wall slide  Terminal knee extension  Hamstring & gastrocnemius stretching | 1 |
| 10 Rep.  10 Rep.  10 Rep.  30 second | 3  3  3  3 | SLR + TB  Wall slide + TB  Terminal knee extension + TB  Hamstring & gastrocnemius stretching | 2 |
| 10 Rep.  10 Rep.  10 Rep.  30 second | 3  3  3  3 | SLR + stronger TB  Wall slide + stronger TB  Terminal knee extension + stronger TB  Hamstring & gastrocnemius stretching | 3 |
| 10 Rep.  10 Rep.  10 Rep.  30 second | 3  3  3  3 | SLR + stronger TB  Wall slide + stronger TB  Terminal knee extension + stronger TB  Hamstring & gastrocnemius stretching | 4 |

SLR; Straigth leg raise, TB; Theraband, Rep; Repetition.

| **Repetition/second** | **sets** | **MSI physiotherapy exercises** | **Week** |
| --- | --- | --- | --- |
| 10 Rep.  10 Rep.  10 Rep.  30 second | 3  3  3  3 | clamshell exercise  side SLR  Prone hip extension  ITB stretching | 1 |
| 10 Rep.  10 Rep.  10 Rep.  30 second | 3  3  3  3 | clamshell exercise + TB  side SLR + TB  Prone hip extension + TB  ITB stretching | 2 |
| 10 Rep.  10 Rep.  10 Rep.  30 second | 3  3  3  3 | clamshell exercise + stronger TB  side SLR + stronger TB  Prone hip extension + stronger TB  ITB stretching | 3 |
| 10 Rep.  10 Rep.  10 Rep.  30 second | 3  3  3  3 | clamshell exercise + stronger TB  side SLR + stronger TB  Prone hip extension + stronger TB  ITB stretching | 4 |

**Supplementary 4: Treatment protocol and progression of exercises for the MSI-based treatment group**

SLR; Straigth leg raise, ITB; Iliotibial band, TB; Theraband, Rep; Repetition.

**Supplementary 5: Alignment and movement impairments data form**

|  | | | |
| --- | --- | --- | --- |
| **Physical Examination Item** | **Unit of Measurement** | | |
|  | |  |  |
| Femoral adduction/internal rotation with partial squat | | Yes | No |
| Knee valgus with partial squat | | Yes | No |
|  | | Yes | No |
| Tibia external rotation/abduction with two joint hip flexor length test | | Yes | No |
|  | | Yes | No |
| Tibia external rotation of the swing leg with step up | | Yes | No |
| Femoral adduction and knee valgus with step down | | Yes | No |
| Femoral adduction and knee valgus when rising from sitting to standing | | Yes | No |
|  | |  |  |
